# Modulation of oxidative stress and subsequent induction of apoptosis and endoplasmic reticulum stress allows citral to decrease cancer cell proliferation

**DOI:** 10.1038/srep27530

**Published:** 2016-06-08

**Authors:** Arvinder Kapur, Mildred Felder, Lucas Fass, Justanjot Kaur, Austin Czarnecki, Kavya Rathi, San Zeng, Kathryn Kalady Osowski, Colin Howell, May P. Xiong, Rebecca J. Whelan, Manish S. Patankar

**Affiliations:** 1 Department of Obstetrics and Gynecology, University of Wisconsin-Madison, Madison, WI-53792-6188, USA; 2 School of Pharmacy, University of Wisconsin-Madison, Madison, Wisconsin 53705-2222, USA; 3 Department of Chemistry and Biochemistry, Oberlin College, Oberlin, OH 44704, USA.

## Abstract

The monoterpenoid, citral, when delivered through PEG-b-PCL nanoparticles inhibits *in vivo* growth of 4T1 breast tumors. Here, we show that citral inhibits proliferation of multiple human cancer cell lines. In p53 expressing ECC-1 and OVCAR-3 but not in p53-deficient SKOV-3 cells, citral induces G1/S cell cycle arrest and apoptosis as determined by Annexin V staining and increased cleaved caspase3 and Bax and decreased Bcl-2. In SKOV-3 cells, citral induces the ER stress markers CHOP, GADD45, EDEM, ATF4, Hsp90, ATG5, and phospho-eIF2α. The molecular chaperone 4-phenylbutyric acid attenuates citral activity in SKOV-3 but not in ECC-1 and OVCAR-3 cells. In p53-expressing cells, citral increases phosphorylation of serine-15 of p53. Activation of p53 increases Bax, PUMA, and NOXA expression. Inhibition of p53 by pifithrin-α, attenuates citral-mediated apoptosis. Citral increases intracellular oxygen radicals and this leads to activation of p53. Inhibition of glutathione synthesis by L-buthionine sulfoxamine increases potency of citral. Pretreatment with N-acetylcysteine decreases phosphorylation of p53 in citral-treated ECC-1 and OVCAR-3. These results define a p53-dependent, and in the absence of p53, ER stress-dependent mode of action of citral. This study indicates that citral in PEG-b-PCL nanoparticle formulation should be considered for treatment of breast and other tumors.

Citral, a pure mixture of the two monoterpenoid isomers, neral and geranial, is a widely used food additive approved by the US Food and Drug Administration as generally safe for human and animal consumption^1^,^2^. *In vitro* studies have reported on the ability of citral to induce cell death of breast cancer as well as leukemia cells^3^,^4^. In a model for chemically-induced skin cancer, chronic application of citral resulted in a decrease in the number of animals developing tumors^5^. Additionally, the number of tumors per mice and tumor volume in the citral treated cohort was significantly less than untreated controls.

We have previously demonstrated that monoterpene extract of ginger rhizomes is enriched in neral and geranial (components of citral) and is a potent suppressor of cancer cell proliferation^6^. Recently, we also demonstrated that a nanoparticle formulation of citral is effective in controlling growth of subcutaneously implanted 4T1 mouse breast tumors. In this same study we showed that of the two isomers, geranial was more effective in controlling *in vivo* tumor growth. Retro-orbital injection of nanoparticles containing geranial at three doses of 80 mg/kg resulted in approximately 92% reduction in tumor volume as compared to controls that received unloaded nanoparticles^7^.

In these *in vivo* experiments, while there was significant reduction in tumor volume, even high doses of nanoparticles loaded with citral, neral or geranial did not cause noticeable toxicity in the animals^5^,^7^. Overall, all of these previous studies have suggested that citral and its constituents, neral and geranial, be considered as cytotoxic agents for the treatment of solid tumors. A major hurdle in the use of citral as an anti-cancer therapeutic is the lack of understanding of the mechanism by which this monoterpenoid induces cancer cell death. While previous reports have demonstrated an increase in cleaved caspase-3 in cancer cells treated with citral^3^,^4^, the upstream mechanisms that result in the activation of this apoptosis-mediating caspase in these experiments are unclear.

The current study was therefore designed to investigate the mechanism of action of citral and to gain insight into molecular phenotype of cancer cells that make them susceptible to citral-mediated apoptosis. Data obtained in our study demonstrate that treatment with citral causes an increase in intracellular oxygen radicals and the resulting oxidative stress is the initiating and essential factor that leads to decreased proliferation and cancer cell death. Additionally, we also demonstrate that citral-induced oxidative stress activates p53 to induce apoptosis and in cancer cells lacking this tumor suppressor, inhibits proliferation by inducing endoplasmic reticulum stress.

## Results

### Inhibition of *in vivo* tumor growth following administration of citral-encapsulated PEG-b-PCL micelles

**Figure 1:**
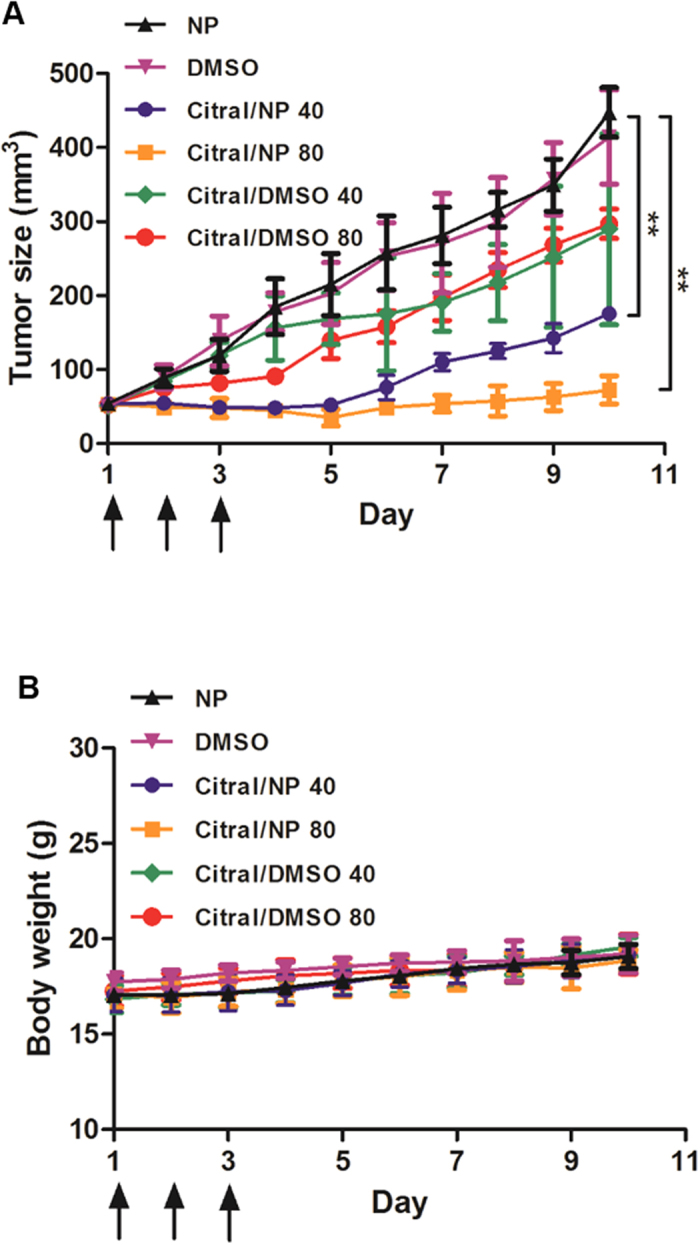
PEG-b-PCL micelle-encapsulated citral inhibits *in vivo* tumor growth. When the 4T1 tumor reached 50 mm^3^, the mice (5 per group) received three retro-orbital injections on days 1, 2 and 3 (indicated by arrows) of: NP vehicle, 2.5% DMSO, citral/DMSO or citral/NP (PEG-PCL micelle-encapsulated citral). Two concentrations of citral, 40 or 80 mg/kg, were tested. *A*, The control NP and DMSO treatments did not inhibit tumor growth. Compared to controls, citral/DMSO 40 and citral/DMSO 80 were similarly effective in inhibiting growth of the aggressive 4T1 tumors (p < 0.01). However, there was significantly improved tumor regression with citral/NP at both 40 (p < 0.001) and 80 mg/kg (p < 0.001) by day 10. *B*, the formulations did not generate acute toxicity in animals based on consistent animal weights throughout the treatment period.

Recently^7^, we demonstrated that citral and its constituent isomers neral and geranial, when administered in a nanoparticle micelle formulation, caused significant decrease in growth of 4T1 tumors in autologous BALB/c mice. In this previous study, four injections of the monoterpene formulations were administered every third day after the tumors had attained a size of 50 mm^3^. The high level of tumor inhibition observed in these experiments prompted us to further test the efficacy of the treatment by administering citral over a shorter period of time. Thus, once the 4T1 tumors attained a size of 50 mm^3^, three doses of citral encapsulated PEG-b-PCL micelles (40 and 80 mg/Kg body weight) were administered. Even with this truncated regimen, treatment with 40 and 80 mg/Kg of citral in PEG-PCL micelles resulted in 60 and 85% reduction of tumor growth, respectively, (Fig. 1A). Citral when delivered in the PEG-b-PCL micelle formulation produced higher level of inhibition than when this monoterpene was delivered in dimethyl sulfoxide (DMSO). The treatment with citral was not overtly toxic as we did not detect statistically significant effect on the body weights of animals in the control and test groups (Fig. 1B).

### Citral inhibits proliferation of cancer cell lines

These *in vivo* experiments are promising and we are continuing with our efforts to develop citral-loaded PEG-b-PCL formulations for the treatment of breast and ovarian tumors. For this method to be successful, however, it is important to identify the molecular mechanisms that allow citral to inhibit tumor growth in human cancer cells. Therefore, for the current study, we focused our attention on understanding citral-induced changes that result in decreased proliferation and increased cell death in cancer cells.

**Figure 2:**
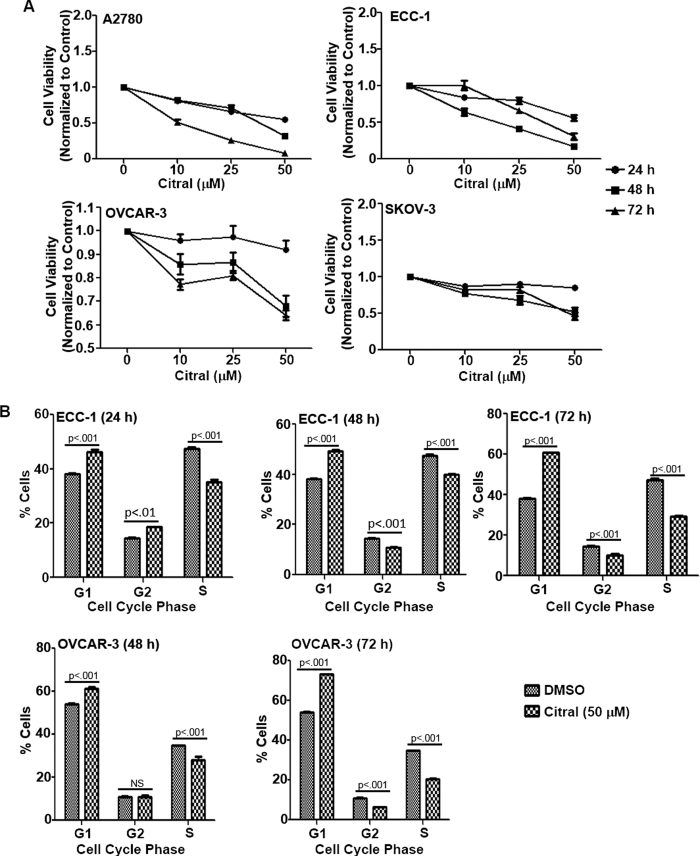
Citral inhibits proliferation and causes cell cycle arrest. *A*, MTT assays were conducted with four cell lines, A2780 (upper left panel), ECC-1 (upper right panel), OVCAR-3 (lower left panel) and SKOV-3 (lower right panel) to determine the anti-proliferative effect of citral. All cell lines were treated with the designated concentrations of citral for 24, 48 and 72 h. After incubation, MTT reagent was added and formazan formation was monitored by reading the absorbance in each well at 570 nm. Each data point is average of eight separate readings and represents three independent replicate experiments. Statistical analyses of the data are provided in Supplementary File 1. *B*, ECC-1 (upper panel) and OVCAR-3 (lower panel) cells were treated with citral (50 μM) for the designated time points. After treatment, cells were fixed, stained with propidium iodide and analyzed by flow cytometry. Results with both cell lines showed an increase in percentage of cells in G1 phase and decrease in cells in the S phase following citral treatment. Each bar is average of three replicates and p values in the bar graph provide statistical significance of these results.

First, we tested the effect of citral on *in vitro* proliferation of a panel of human cancer cell lines. When tested in MTT (3-(4,5-dimethythiazol-2-yl)-2,5-diphenyl tetrazolium bromide) assays, citral inhibited proliferation of the human cancer cell lines A2780, ECC-1, OVCAR-3 and SKOV-3 (Fig. 2A; Supplementary File 1 for statistical analysis). Citral, however did not inhibit the proliferation of RMF-CC, an immortalized human mammary fibroblast cell line^8^ (Supplementary File 2; Supplementary File 1 for statistical analysis). In comparison to citral, the monoterpenes, α-pinene and camphene, showed weak bioactivity in the MTT assays conducted with ECC-1 cells (Supplementary File 3 and for statistical analysis see Supplementary File 1). These results indicate that the anti-proliferative effects demonstrated by citral are specific and not shared by all monoterpenoids.

### Citral causes G1/S cell cycle arrest and induces apoptosis

To test the reason for the decrease in proliferation observed in citral-treated cells, we conducted cell cycle assays. In both ECC-1 and OVCAR-3 cells, citral induced cell cycle arrest in the G1/S phase (Fig. 2B). Cell cycle arrest was observed at all time-points tested. However, cell cycle arrest was not observed in SKOV-3 cells exposed to citral (data not shown).

**Figure 3:**
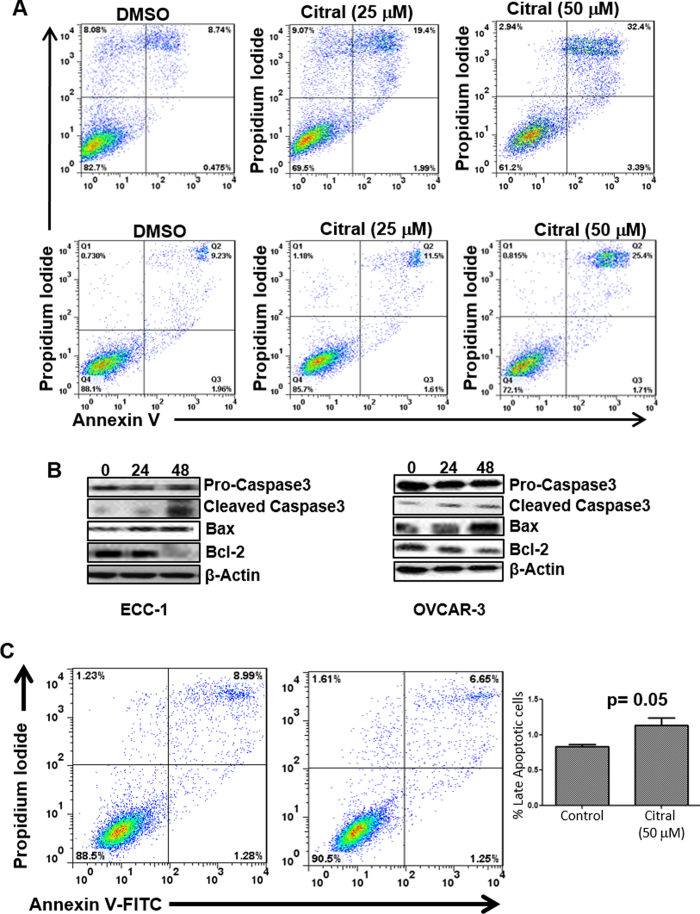
Citral induces apoptosis. *A*, To monitor cell death via apoptosis, ECC-1 (upper panel) and OVCAR-3 (lower panel) cells were treated overnight with designated concentrations of citral. After treatment, cells were labeled with Annexin-V-FITC and propidium iodide. Apoptotic cell death in the cells was monitored by flow cytometry. Data presented is representative of three independent experiments. *B*, Citral induced apoptosis in ECC-1 and OVCAR-3 cells was confirmed by monitoring for cleaved caspase-3 and expression of Bax and Bcl-2 by western blotting. Lysates of ECC-1 and OVCAR-3 cells treated with citral (25 and 50 μM, respectively) for 0, 24 and 48 h were used in the Western blot analysis. Data shown is representative of three independent experiments. *C*, Annexin-V-FITC and propidium iodide staining assays do not show an increase in apoptotic SKOV-3 cells treated for 48 h with 50 μM citral. Representative dot plots are shown. Bar graph is data from three independent experiments.

That SKOV-3 cells were responding differently to citral than ECC-1 and OVCAR-3 cells was also clear in apoptosis assays. In addition to cell cycle arrest, we also observed that treatment with citral resulted in the death of ECC-1 and OVCAR-3 cells via apoptosis. Annexin V assays showed that exposure to citral (50 μM) for 24 h resulted in 25 and 32% apoptosis in OVCAR-3 and ECC-1 cells as compared to 9% in vehicle treated OVCAR-3 and ECC-1cells, respectively (Fig. 3A). Apoptosis in OVCAR-3 (Fig. 3B) and ECC-1 cells (Fig. 3B) was also confirmed by western blot analysis showing an increase in cleaved caspase3 and Bax and a decrease in expression of Bcl-2. To the contrary, increase in annexin V staining was not observed in citral-treated SKOV-3 cells (Fig. 3C).

### Citral-induced ER stress is responsible for decreased proliferation of SKOV-3 cells

While citral did not increase apoptosis or cause cell cycle arrest of SKOV-3 cells, we did observe an increase in staining of these cells by acridine orange, a dye that localizes to the acidic organelles such as the autophagolysosomes (Supplementary File 4). Western blot analysis of citral-treated SKOV-3 cells showed marginal increase in expression of ATG5 and significant increase in the expression of LC3BII (Supplementary File 4). Increase in LC3BII expression was also corroborated by a flow cytometry-based assay for this protein (Supplementary File 4). These observations suggested that citral may be inducing autophagy in SKOV-3 cells. Interestingly, the 4T1 tumors excised from mice treated with citral encapsulated PEG-b-PCL micelles (Fig. 1) also showed an increase in the expression of the autophagy markers LC3B-II and Atg5 (Supplementary File 4).

**Figure 4:**
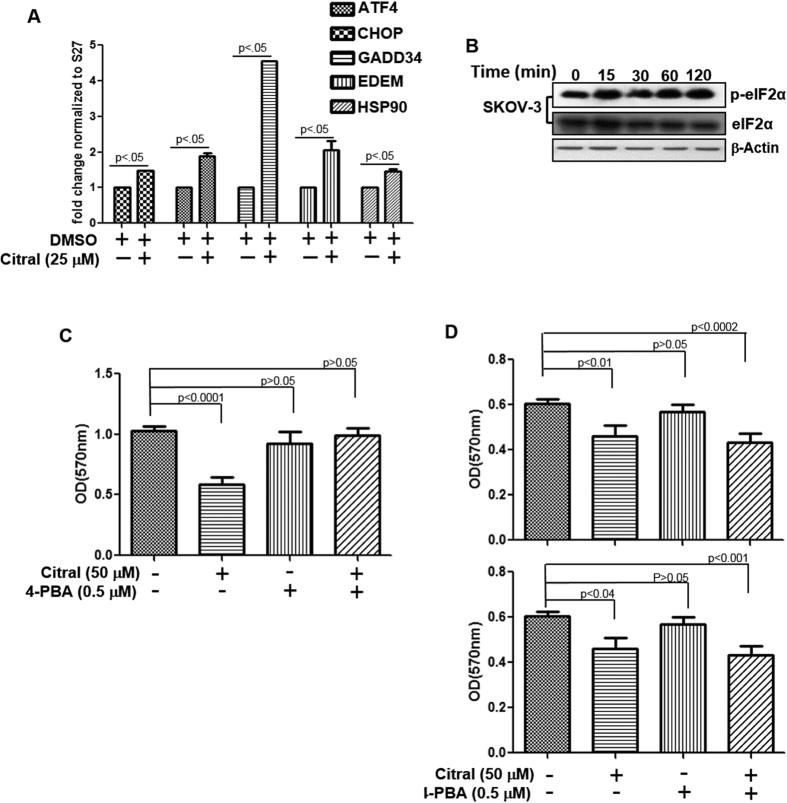
Citral inhibits proliferation of SKOV-3 cells by inducing ER stress. *A*, After incubating SKOV-3 cells in media or media containing citral for 6 h, cells were lysed and RNA was isolated. qPCR was used to monitor expression of the ER stress markers. *B*, ER stress in citral-treated SKOV-3 cells was confirmed by western blotting to monitor phosphorylation of eIF2α. *C,* SKOV-3 and *D*, OVCAR-3 (upper panel) and ECC-1 (lower panel) cells were treated with citral for 72 h in the presence or absence of 4-PBA. MTT assays were performed to determine effect of the treatments on proliferation of the cells. Each bar in this plot is mean of eight readings and is representative of data obtained from two independent experiments.

We further observed that when SKOV-3 cells were treated with citral (25 μM) there was significant increase in the expression of the ER stress markers ATF4, CHOP, GADD34, EDEM and HSP90 (Fig. 4A). Further evidence of ER stress occurring in citral treated cells was obtained through western blotting experiments showing an increase in phospho-eIF2α in SKOV-3 cells (Fig. 4B). An increase in expression of phospho-eIF2α was also observed in ECC-1 (Supplementary file 5). ER stress is likely a common event that occurs irrespective of whether citral is able to inhibit proliferation via apoptosis-dependent or independent mechanism.

4-Phenyl butyric acid (4-PBA) alleviates protein misfolding by serving as a molecular chaperone and thereby relieves ER stress^9^,^10^,^11^. When SKOV-3 cells were treated with citral in the presence of 4-PBA, the inhibitory effect of citral was reversed (Fig. 4C). However, 4-PBA did not alleviate citral-mediated inhibition of ECC-1 and OVCAR-3 cell proliferation (Fig. 4D).

### Citral treatment activates p53 in ECC-1 and OVCAR-3 cells

**Figure 5:**
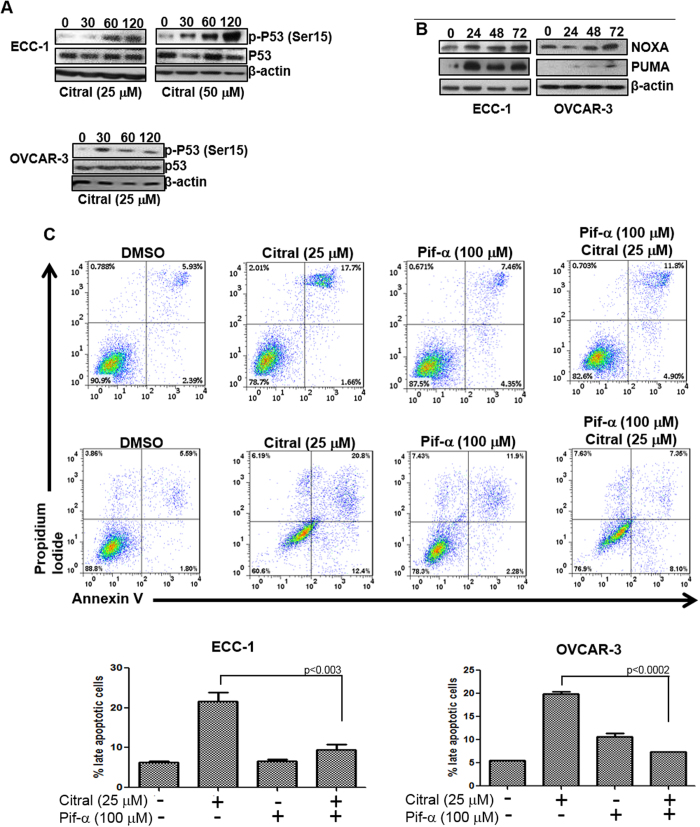
Citral-mediated activation of p53 results in apoptotic cell death. *A*, ECC-1 and OVCAR-3 cells were treated with citral (concentration denoted below each western blot) for the designated time points. After treatment, cell lysates were probed for phosphorylation status of Serine-15 (Ser15) of p53 and for total p53. *B*, ECC-1 and OVCAR-3 cells were treated for the designated time points with citral (25 μM). Cell lysates were monitored for expression of p53-responsive genes PUMA and NOXA in both cell lines. *C*, ECC-1 and OVCAR-3 cells were treated with citral in the presence or absence of the p53 inhibitor pifithrin-α (Pif-α). After incubation for 24 h, apoptosis in the cells was determined by annexin V-FITC and propidium iodide staining. Cells were monitored using flow cytometry. Dot plots in the upper and lower panels are representative data obtained from ECC-1 and OVCAR-3 cells, respectively. The bar charts at the bottom show percentage of apoptotic cells after treatment from three independent experiments.

Experiments with 4-PBA indicated that although citral decreased proliferation of SKOV-3 cells via induction of ER stress, in the case of ECC-1 and OVCAR-3 the mechanism of action of citral was distinct. As shown in Fig. 3B, treatment with citral resulted in increase in expression of Bax, a gene that is transcriptionally regulated by the tumor suppressor p53^12^,^13^. SKOV-3 cells are p53 deficient, whereas, both ECC-1 and OVCAR-3 express mutated p53^14^,^15^,^16^,^17^. We therefore hypothesized that activation of p53 by citral could be resulting in apoptosis of ECC-1 and OVCAR-3, whereas, deficiency of this tumor suppressor in SKOV-3 would explain the lack of cell death via the apoptotic pathway. This hypothesis was supported by the data that treatment of ECC-1 and OVCAR-3 cells with citral resulted in increase in phosphorylation of the serine-15 residue of p53 in both these cell lines (Fig. 5A). Phosphorylation of Serine-15 residue of p53 is known to activate this tumor suppressor and could explain the increase in expression of Bax as observed in Fig. 2B. Additional proof, that citral activates p53 in ECC-1 and OVCAR-3 cells was obtained by western blot analysis showing an increase in expression of PUMA and NOXA, two pro-apoptotic genes that are also transcriptionally regulated by p53 (Fig. 5B). Furthermore, pifithrin-α, an inhibitor of p53^6^,^18^, abrogated (by 3-4-fold) citral induced apoptosis in ECC-1 and OVCAR-3 cells (Fig. 5C).

### Citral decreases the cellular levels of Glutathione (GSH) and increases intracellular oxygen radicals

With results from Fig. 5 demonstrating that activation of p53 by citral was essential for apoptotic cell death of ECC-1 and OVCAR-3 cells, we next examined the upstream events responsible for activation of this tumor suppressor.

**Figure 6:**
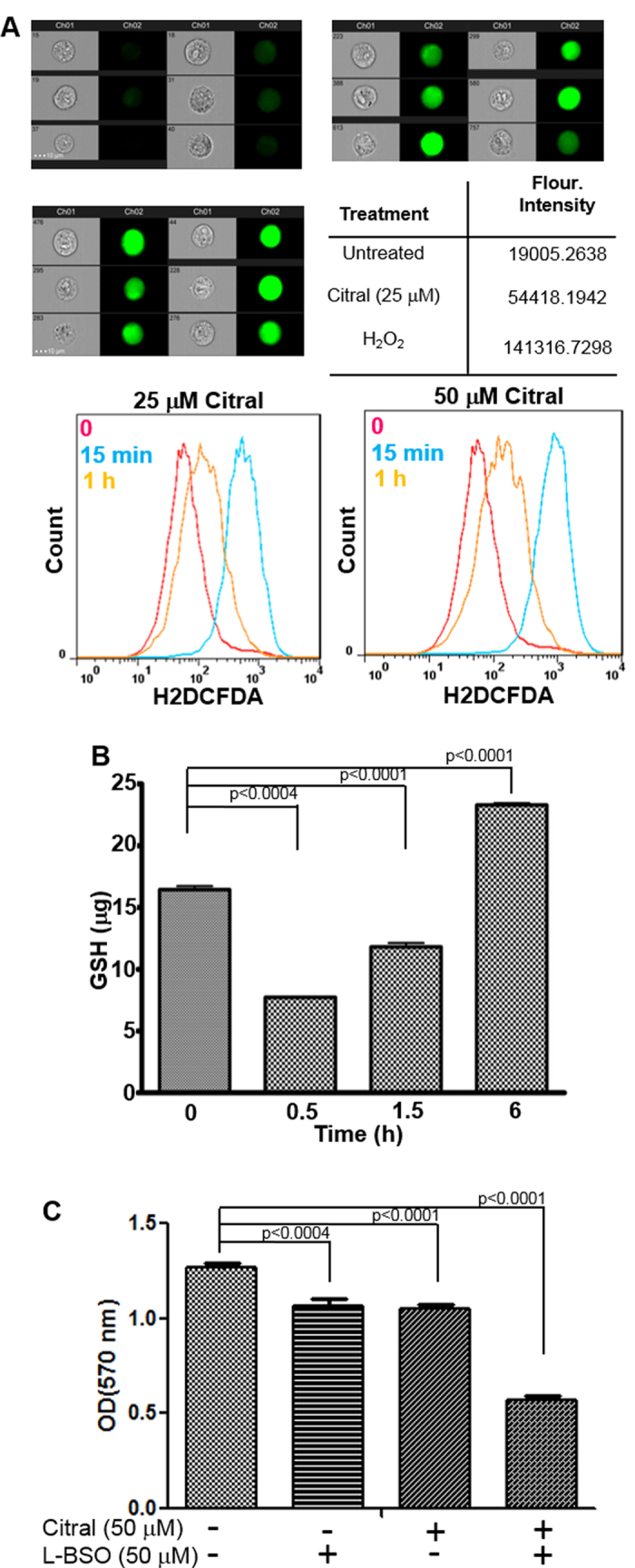
Citral-induces oxidative stress. *A*, To monitor changes in intracellular oxygen radicals, ECC-1 cells were labeled with the oxygen radical sensor, H2DCFDA, prior to treatment with citral. Increase in oxygen radicals was monitored by imaging cytometry (upper and middle panels) and flow cytometry (lower panel). Imaging cytometry data is shown for six representative cells out of 10,000 total events analyzed. Data shown is representative of two separate experiments. Bright field and fluorescent images for each treatment are shown. Results from cells treated with media (upper left), 25 μM citral (upper right) and hydrogen peroxide (middle left) are shown. The table (middle right) shows mean fluorescence intensity of the 10,000 total evens for each condition tested. To determine changes in intracellular oxygen radical levels over time, H2DCFDA-labeled ECC-1 cells were monitored by flow cytometry after treatment for 0, 15 min and 1 h with 25 and 50 μM citral (bottom left and right panels). Each histogram is representative of three independent experiments. *B*, ECC-1 cells were treated with citral (50 μM). At the designated time points, the cells were lysed to measure levels of GSH. Data shown is representative of two independent experiments, each conducted in duplicate. *C*, ECC-1 cells were treated with citral and L-BSO as shown. Proliferation of the cells was determined at 72 h using MTT assay.

Several studies have demonstrated that oxygen radicals contribute to ER stress^19^ and activate p53^20^,^21^. We investigated the effect of citral treatment on reactive oxygen radical production in cancer cells. ECC-1 cells were incubated with the oxygen radical sensing dye 2′,7′ di-chloro-dihydro-fluorescein-diacetate (H2DCFDA) and subsequently treated with citral. Imaging cytometry conducted after 15 min exposure of the cells to citral showed a strong increase in H2DCFDA fluorescence (Fig. 6A). Flow cytometry experiments at defined time points confirmed increase in intracellular oxygen radicals as early as 15 minutes after treatment with citral (Fig. 6A). Increased levels of oxygen radicals were also detected after 1 h exposure to citral. However, the level of oxygen radicals at the 1 h timepoint was lower than that observed after 15 min treatment with citral (Fig. 6A). Similar results were obtained when oxygen radicals were monitored in OVCAR-3 and SKOV-3 cells (data not shown).

Citral is a α,β-unsaturated aldehyde which participates in a Michael addition reaction with sulfhydryl groups of GSH^19^,^20^ and cysteine residues of proteins (Supplementary File 6). The effect of citral on the intracellular level of GSH was therefore monitored. ECC-1 cells were treated with 50 μM citral for 0.5, 1.5 and 6 h following which the cells were lysed and concentration of GSH was measured. A rapid decline in intracellular GSH was observed as early as 0.5 h after treatment with citral (Fig. 6B).

### Inhibition of GSH synthesis increases the activity of citral

Although citral treatment resulted in a significant decline in intracellular level of GSH at 0.5 h, by 1.5 h, when intracellular oxygen radicals had also subsided (Fig. 6A,B), a recovery of GSH was observed (Fig. 6B). At 6 h after exposure to citral, GSH levels were significantly higher than those observed at baseline (Fig. 6B). This increase in GSH is likely due to its de novo synthesis as the cancer cell responds to citral-induced oxidative stress.

Addition of cysteine to the gamma carbon of glutamate is the rate limiting step of GSH synthesis and is catalyzed by the enzyme γ-glutamylcysteine ligase (γ-GCL). L-Buthionine sulfoximine (L-BSO) is an inhibitor of γ-GCL^21^. Combined treatment of ECC-1 cells for 24 h with citral and L-BSO resulted in a synergistic increase in the potency of each agent as demonstrated by approximately 2-fold increase in the attenuation in proliferation of ECC-1 cells (Fig. 6C).

### Induction of intracellular oxygen radicals by citral activates p53 and triggers apoptosis

**Figure 7:**
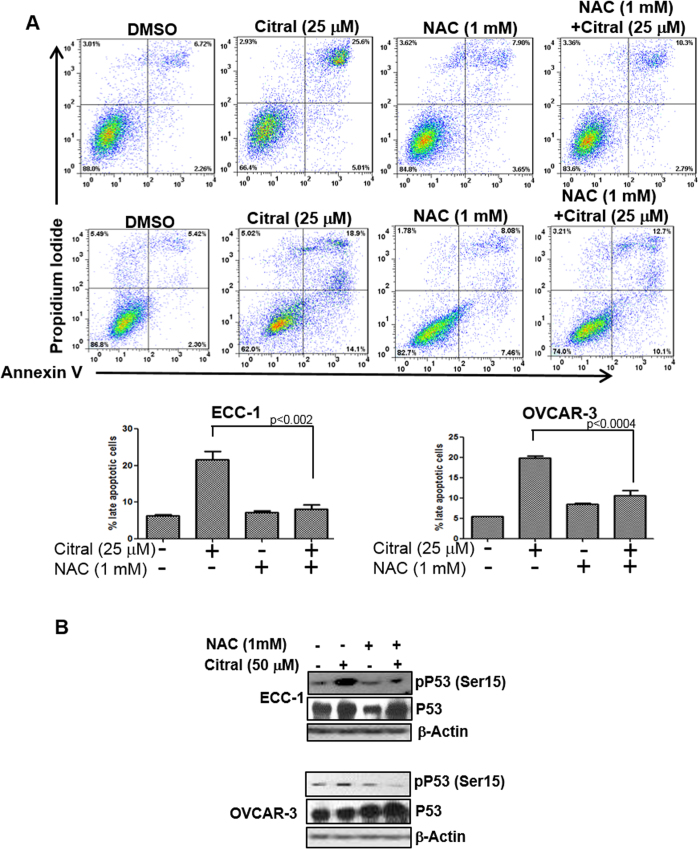
Citral-induced oxidative stress is responsible for apoptosis and p53 activation. *A*, to determine the contribution of oxygen radicals in citral-mediated cell death, ECC-1 (upper panel) and OVCAR-3 cells (middle panel) were incubated with citral in the presence or absence of NAC. After treatment for 24 h, the cells were labeled with Annexin V-FITC and propidium iodide and cell death was determined by flow cytometry. Dot plots in upper and lower panels are representative data obtained using ECC-1 and OVCAR-3 cells, respectively. The bar graphs in the bottom panel are data obtained from three independent experiments with ECC-1 and OVCAR-3 cells. *B*, Evidence confirming that citral-induced oxygen radicals were required for activation of p53 was obtained by treating ECC-1 and OVCAR-3 cells with citral in the presence or absence of the radical scavenger, N-acetylcysteine (NAC). After treatment for 30 min, cell lysates were obtained and probed to assess Serine-15 (Ser15) phosphorylation of p53.

The synergistic action of citral and L-BSO suggested that increase in intracellular oxygen radicals was an important component of the bioactivity of this monoterpenoid. In cells treated with citral in the presence of the oxygen radical scavenger, N-acetylcysteine (NAC), apoptosis was reduced to 8% and 10% in ECC-1 and OVCAR-3 cells, respectively (Fig. 7A). In comparison, when ECC-1 and OVCAR-3 cells were treated only with citral (50 μM), the percentage of cells undergoing apoptosis was between 20-25% (Fig. 7A). Finally, when ECC-1 and OVCAR-3 cells were pre-treated with NAC, phosphorylation of p53 on the serine-15 residue in response to citral was significantly attenuated (Fig. 7B). Collectively, these results demonstrate a central role for oxidative stress in citral mediated regulation of cancer cell proliferation.

## Discussion

Results presented in the current study demonstrate that citral, because of its anti-proliferative effects, should be considered a candidate for chemoprevention and chemotherapy. In the majority of the cell lines tested, the IC50 of citral was between 20–50 μM. In comparison, previous studies on this cell line panel conducted by our group have shown that cisplatin has an IC50 between 5–15 μM.

Previous reports have demonstrated anti-cancer effects of citral^3^,^22^,^23^,^24^,^25^. Our study, provides understanding of the molecular mechanisms that enable citral to inhibit cancer cell proliferation. Being a relatively small and hydrophobic molecule, citral diffuses through the cell membranes. Citral is a strong electrophile because of its α,β-unsaturated carbonyl functionality^20^. Therefore, mammalian cells readily metabolize this xenobiotic molecule^26^. GSH conjugation, via non-enzymatic (direct chemical reaction) and enzymatic (via Glutathione S-transferase, GST) reactions, is an important step in intracellular metabolism of citral^19^. As a result, it is tempting to conclude that citral may be acting as a chemical sink and hence causing a decrease in intracellular GSH levels. However, intracellular GSH levels are typically between 0.5–10 mM^27^. Since in our assays, cancer cells were treated with 10–50 μM citral, the stoichiometry of the reaction suggests that other mechanisms be considered to explain the rapid loss of intracellular GSH following citral treatment.

The ratio of GSH to its oxidized derivative, GSSG, is tightly regulated and approximately 90% is found in the reduced, GSH, form. Minor changes in the GSSG:GSH ratio may cause oxidative stress. Additionally, GSH conjugates with compounds containing α,β-carbonyl functionality act as inhibitors of GST^28^. Although the citral-GSH conjugate is a weak inhibitor of GST, the potential effect of this compound on γ-GCL activity is unknown. Another possibility to consider is that citral may directly conjugate with critical thiol groups of enzymes that are important for GSH synthesis and thereby produce an immediate and pronounced effect on the intracellular levels of this important anti-oxidant compound.

[6]-Shogaol, a phenolic natural product, inhibits proliferation of cancer cells by reducing intracellular GSH^28^,^29^,^30^. It is important to note that [6]-shogaol also contains the α,β-unsaturated carbonyl functional group that our data predicts to be an important feature for the bioactivity of monoterpenoids. Studies on natural products that contain this functional group should therefore consider intracellular GSH depletion as a potential mechanism of action to explain the anti-cancer effects of these compounds.

Citral-induced increase in intracellular oxygen radicals triggers activation of p53 and subsequent apoptosis of the cells. OVCAR-3 cells express mutant p53^16^. Therefore, studies with this ovarian cancer cell line suggest that some p53 mutants may retain the capacity to induce apoptosis when activated by cellular stress or citral may be reactivating at least a subset of p53 mutants to induce apoptosis. However, apoptosis is not essential for citral to inhibit the proliferation of cancer cells. SKOV-3 cells are p53-deficient but citral is able to inhibit the proliferation of these cells even though apoptosis is not detected. Since 4-PBA is able to overcome citral-induced suppression of proliferation in SKOV-3 cells, ER stress is likely the mechanism of action in cells that lack p53.

The 4T1 cells, similar to SKOV-3, are p53 deficient and as shown by the data in Supplementary File 4, citral induces an increase in the autophagy markers LC3B-II and Atg5. These results therefore suggest that in the absence of p53, the decreased proliferation mediated by citral is likely due to ER stress and autophagy. Our ongoing experiments are focused on determining if necrotic cell death is initiated by citral in p53 deficient tumors and the potential role of autophagy in such a type of cell demise.

Although citral induces an initial decrease in intracellular GSH, the cancer cells compensate for this loss by increasing de novo synthesis of GSH (Fig. 6A). This observation provides two separate opportunities to develop distinct classes of agents that can be used for chemoprevention as well as chemotherapy. By triggering de novo synthesis of GSH, citral could act as an anti-oxidant and hence serve an important purpose in chemoprevention. We are investigating nanoparticle-based delivery^7^ as well as chemical modifications as strategies to improve the pharmacokinetic profile of citral with the primary goal to develop a novel citral-based strategy for chemotherapy. The nanoparticle-citral formulations have shown increased anti-tumor efficacy in *in vitro* as well as *in vivo* experiments^7^. Targeting citral-based therapeutics to tumors that have retained the ability of p53 to induce apoptosis will be an important approach to consider in future translational and clinical studies.

## Methods

### Reagents and Cell Lines

DMEM (Dulbecco’s Modification of Eagle’s Medium), RPMI-1640, Hanks Balanced Salt Solution (HBSS), and Dulbecco’s Phosphate Buffered Saline (DPBS) were from Cellgro (Manassas, VA). SuperSignal West Dura Extended Duration Substrate, RIPA buffer and Protease Inhibitor Cocktail were from ThermoFisher Scientific (Waltham, MA). All primary and secondary antibodies were from Cell Signaling Technology (Beverly, MA) and Jackson ImmunoResearch Laboratories (West Grove, PA), respectively. FITC-Annexin V Apoptosis Detection kit was purchased from BD Pharmingen (San Diego, CA). Cancer cell lines used in this study were purchased from ATCC. The immortalized human mammary fibroblast cell line was a kind gift from Dr. Andreas Friedl (UW-Madison).

### Cell Line Authentication

All cell lines were purchased from ATCC (Manassas, VA) and maintained in the recommended culture media. Experiments with majority of the cell lines were conducted within six months to one year of their purchase from ATCC. The cell lines not fitting this criterion were validated by Single Tandem Repeat (STR) analysis (Genetic DNA laboratory Inc. Burlington, NC). Cell line authentication by STR analysis was conducted not more than six months prior to their use in the reported experiments.

### Study approvals

Experiments performed in mice were approved by the Animal Care and Use committee of the University of Wisconsin-Madison. All experiments were conducted on established human cancer cell lines. Biological materials from healthy human subjects of patients was not used in the studies. All of experiments were approved by the Institutional Biosafety Committee of the University of Wisconsin-Madison and followed all of the regulatory guidelines of this institution.

### Citral preparation used in the current study

Citral is a pure mixture of neral and geranial. The citral preparation used in our studies was a 1:2 mixture of neral and geranial as demonstrated by liquid chromatography with ultraviolet absorbance detection (Supplementary File 6).

### Preparation of citral-encapsulated PEG-b-PCL micelles

The PEG-b-PCL (block sizes of 5000:10000, Mw/Mn 1.26) micelles were prepared as reported previously^7^,^31^. The diblock polymer PEG-b-PCL (0.5 mM) was dissolved in acetone (1 ml), then 1 ml of ddH_2_O was quickly added to the polymer suspension under vigorous stirring. Acetone was removed by evaporation, micelles were centrifuged at 10,000 rpm for 5 min, and passed through a 0.2 μm nylon syringe filter to remove aggregates.

Citral-loaded micelles (citral/NP) were prepared by first dissolving the drug with the polymer in acetone and then adding ddH_2_O. The drug spontaneously loads into the micelle during self-association of the polymer chains. Micelles were centrifuged and passed through a 0.2 μm nylon filter as before to remove un-encapsulated drug and/or aggregates.

Particle size was characterized at 25 °C using a Zeta sizer Nano-ZS (Malvern Instruments, UK) equipped with a He-Ne ion laser (λ = 633 nm). The hydrodynamic diameters of samples were obtained by cumulant method and are reported as Z-average diameters.

The concentration of citral in the micelles was measured by reverse-phase (Symmetry Shield RP18, Waters Corp.) HPLC^31^,^32^ with monitoring conducted by UV detection at 240 nm (the diblock polymer PEG-b-PCL does not absorb at this wavelength). The mobile phase used was a cocktail of 40% acetonitrile and 60% water containing 0.1% acetic acid. Drug loading and encapsulation efficiency were calculated using the following equations:


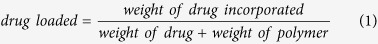



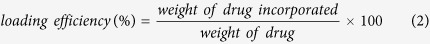


### Effect of citral on *in vivo* growth of 4t1 tumors

Approximately 1 × 10^6^ 4T1 cells were inoculated subcutaneously into the right flanks of anesthetized female BALB/c mice (n = 5 for each control and treatment groups). When the tumor size reached about 50 mm^3^, 200 μl of the formulation (citral/DMSO, citral/NP, DMSO or empty NP) was injected into animals at two final concentration of citral (40 or 80 mg/kg) on days 1, 2 and 3. The tumor size and body weights of animals were monitored daily for 10 days.

### Cell Proliferation Assays

Effect of citral and other terpenoids on the proliferation of cancer cell lines was determined by the 3-(4,5-dimethythiazol-2-yl)-2,5-diphenyl tetrazolium bromide (MTT) uptake method^33^,^34^. Cancer cells in 96 well plates (2,500-5,000 cells/well) were treated with the test compounds at 37 °C in a 5% CO_2_ environment for 24 h, 48 h and 72 h. After incubation, MTT (20 μl of 50 μg/ml stock solution) was added to each well and the plates were incubated at 37 °C for additional 3 h. DMSO (100 μl) was added to each well to dissolve the formazan crystals and absorbance in each well was determined at 570 nm on a Spectra MAX 190 (Molecular Devices, Sunnyvale, CA) plate reader.

### Annexin V assays

Apoptosis in cells treated with citral or other agents was determined by flow cytometry as described^6^. Apoptotic cells were stained with Annexin V-FITC and propidium iodide. The stained cells were analyzed by flow cytometry and data was analyzed using FloJo software (Ashland OR).

### Western blotting

Cells plated in 10 cm plates were treated with citral for 24, 48 or 72 h, washed with ice cold PBS and lysed in RIPA buffer containing protease inhibitor cocktail. Protein concentration was measured using BCA protein assay kit (ThermoFisher, MA). Lysates equivalent to 25 μg of total protein were separated on a 12.5% gel, and blotted onto PVDF membrane. The membranes were probed with primary and secondary antibodies and WestDura or WestFemto reagents (BioRad, CA) were used for detection.

### Measurement of intracellular oxygen radicals

Cells were incubated with 10 μM 2′,7′ di-chloro-dihydro-fluorescein-diacetate (H-DCFDA, Molecular Probes, OR) for 30 min at 37 °C, followed by treatment with citral. The cells were washed with PBS, harvested and ROS content was measured as the fluorescent DCF product on Imagestream II (EMD Millipore, Darmstadt, Germany) imaging cytometer or FACSCalibur (BD Biosciences, CA) flow cytometer. For Imagestream, data was acquired using INSPIRE V.200.1.388.0acquisition software, using Channel1 for Bright Field and Channel 2 (533/55 filter) for Green fluorescence. The images were acquired at a magnification of 40X with 488 illumination of 5mW and 785 illumination at 1.72 mW. The gating was done on single cells, in focus and maximum fluorescence in the positive control sample and same gate was applied to the control and test samples. Results from imaging cytometry were analyzed by using the proprietary IDEAS V 6.1.303.0 analysis software package and FACSCalibur flow cytometry data was analyzed using FlowJo software (Ashland, OR).

### Treatment of cells with N-Acetylcysteine (NAC)

Cells in 10 cm plates were pretreated with 1 mM NAC for 30 min, followed by overnight treatment with 25 μM citral. Cells were monitored for apoptosis by flow cytometry and by western blotting to determine effect of the treatment on p53 phosphorylation.

### Glutathione assay

ECC1 cells were plated in 6 well plates, treated with 25 μM citral for 30 min, and 1.5, 6 and 18 h were washed, scraped and sonicated in 1 ml cold phosphate buffer pH 7, containing 1 mM EDTA. The homogenate was centrifuged (10,000Xg, 15 min at 4 °C), supernatant was removed and deproteinized by adding equal volume of *m*-phosphoric acid. The mixture was allowed to stand at room temperature for five min and centrifuged (2000Xg, 2 min). Glutathione (GSH) in the supernatant was measured using the protocol provided by the manufacturer (Cayman Chemical, Ann Arbor MI).

### Description of pifithrin-α blocking

Cells in 10 cm plates were pretreated with 100 μm pifithrin-α for 2 h, followed by overnight treatment with citral. Cells were washed, harvested and stained for apoptosis using AnnexinV FITC and propidium iodide (BD Biosciences, CA).

### Acridine Orange assay

SKOV-3 cells in six well plates were treated with citral (2.5 μg/ml) for 72 h and then stained with acridine orange (final concentration of 1 μg/ml for 15 min), washed with PBS, harvested by trypsinization, analyzed on a FACScan cytometer and data was analyzed using CellQuest software.

### Monitoring gene expression by qPCR

After treatment with citral for 2, 4 or 6 h, SKOV3 cells were washed and lysed in Trizol and RNA was extracted. RNA was reverse transcribed to cDNA using Omniscript cDNA kit (Qiagen, CA) and qPCR was performed using primers purchased from SA Biosciences, CA. SYBRgreen mastermix was from BioRad, CA and qPCR reactions were conducted on CFX96 Real Time PCR machine.

## Additional Information

**How to cite this article**: Kapur, A. *et al.* Modulation of oxidative stress and subsequent induction of apoptosis and endoplasmic reticulum stress allows citral to decrease cancer cell proliferation. *Sci. Rep.* **6**, 27530; doi: 10.1038/srep27530 (2016).

## Supplementary Material

Supplementary Information
