# Supplementary material for: Modulation of oxidative stress and subsequent induction of apoptosis and endoplasmic reticulum stress allows citral to decrease cancer cell proliferation
Source: Sci Rep. 2016 Jun 8;6:27530. doi: 10.1038/srep27530 (PMC4897611; doi:10.1038/srep27530)
Supplement: Supplementary Information [file srep27530-s1.doc]

**Modulation of oxidative stress and subsequent induction of apoptosis and endoplasmic reticulum stress allows citral to decrease cancer cell proliferation**

Arvinder Kapur, Mildred Felder, Lucas Fass, Justanjot Kaur, Austin Czarnecki, Kavya Rathi, San Zeng, Kathryn Kalady Osowski, Colin Howell, May P. Xiong, Rebecca J. Whelan, Manish S. Patankar


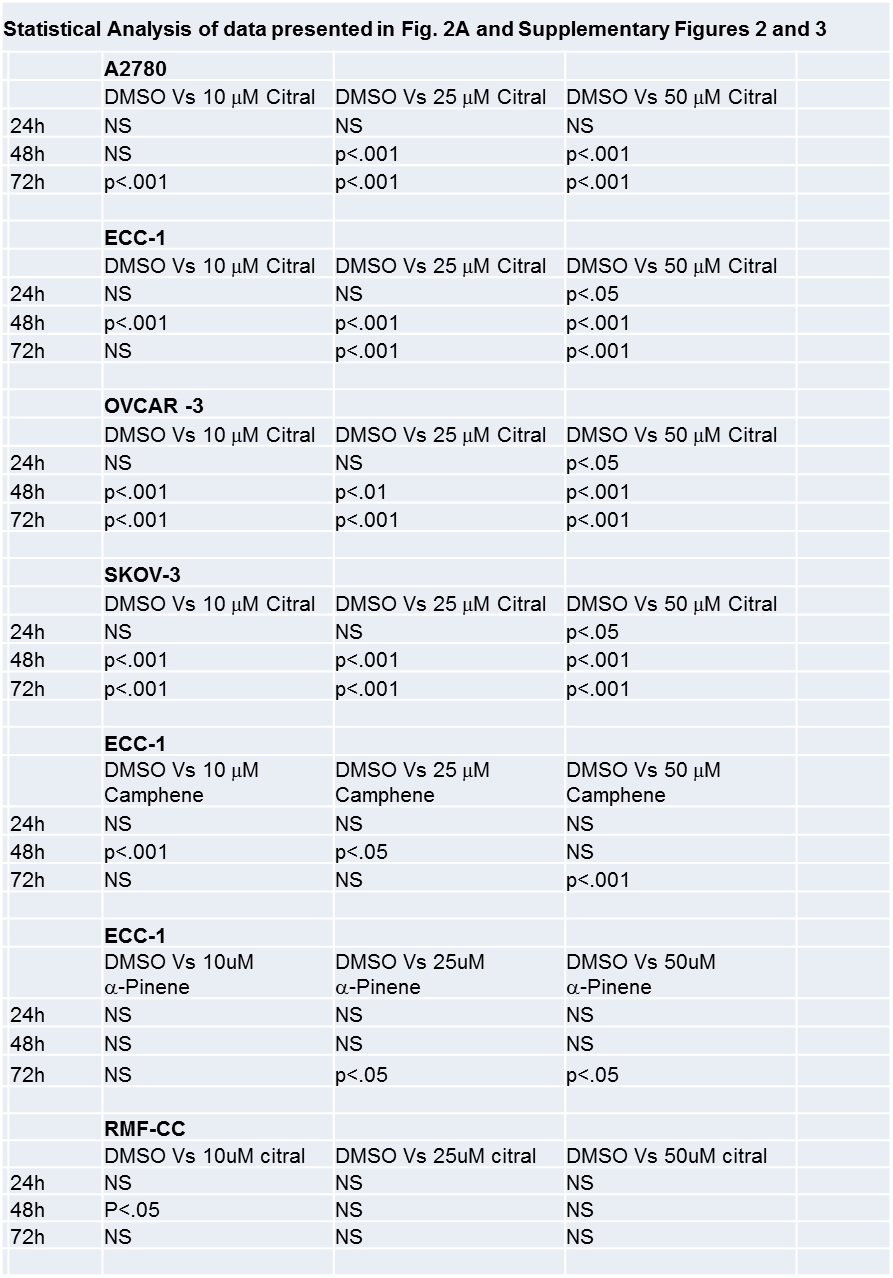


**Supplementary File 1**

**Supplementary File 2**


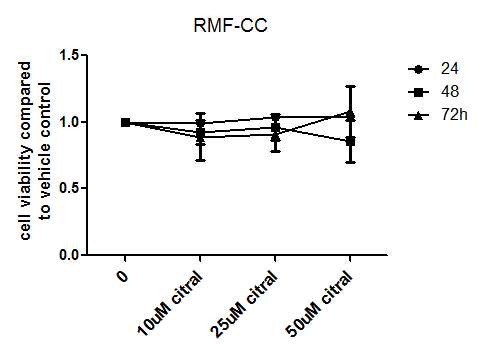
//////

Citral does not inhibit proliferation of the immortalized human mammary fibroblast cell line. Cell proliferation was determined using MTT assay.


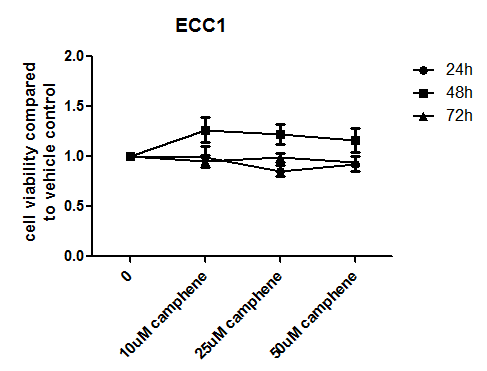

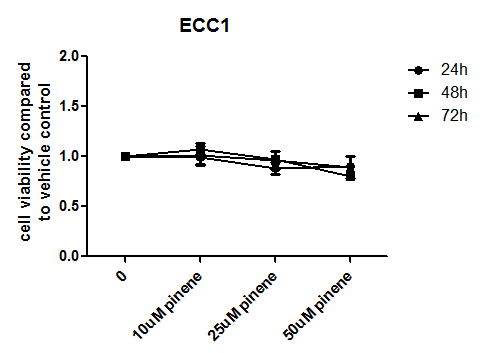


**Supplementary File 3**

Cell proliferation assay (MTT) was conducted to test the effect of monoterpenes camphene and α-pinene on ECC-1 cells. Each point is a mean of three independent experiments with 8 replicates in each experiment.

**0**

**10**

**25**

**50**

**Camphene (μM)**

**0**

**10**

**25**

**50**

**α-Pinene (μM)**


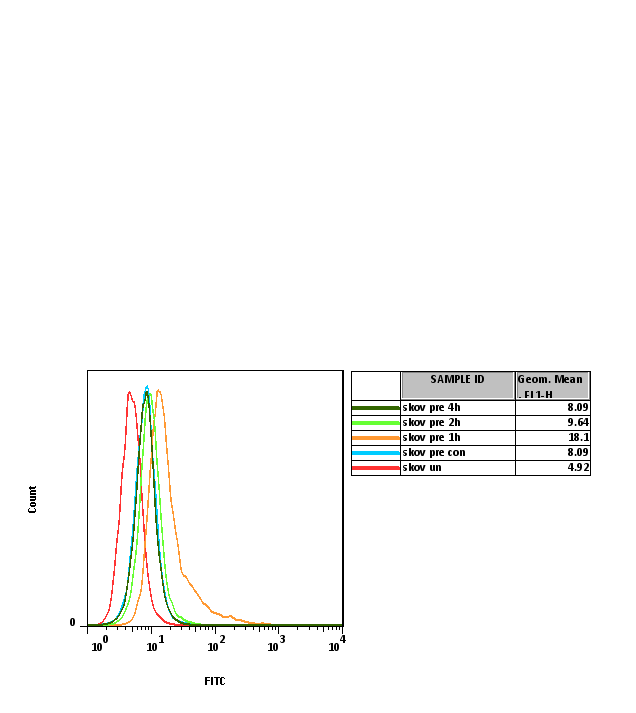


Unlabeled

0 min

1 h

2 h

4 h

**Count**

**H2DCFDA**

**C**


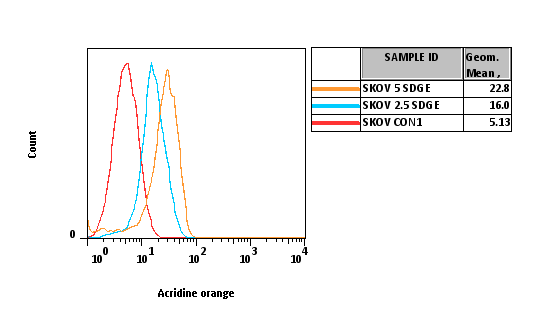


**DMSO**

**Citral (25μM)**

**Citral (50μM)**

**Acridine Orange**

**Count**

**A**


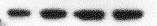


**ATG5**

**LC3B II**


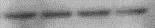


**β-Actin**

**0**

**24**

**48**

**72**


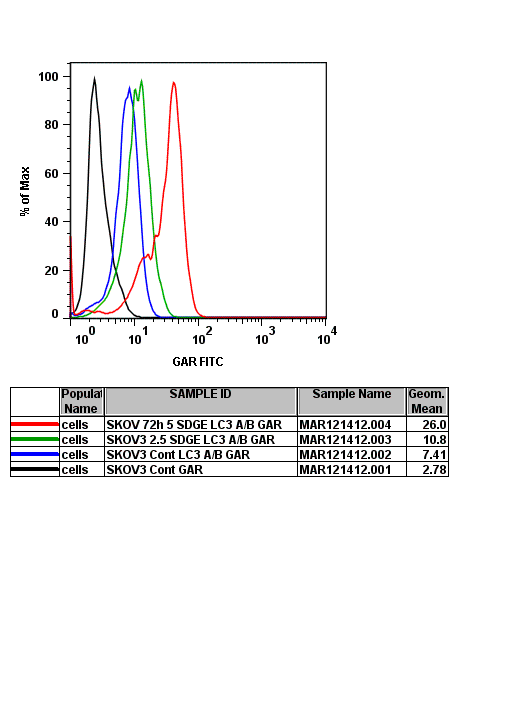


**Secondary Ab**

**DMSO**

**Citral (25μM)**

**Citral (50μM)**

**LC3B**

**% of Max**


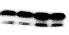


**LC3B I**

**Time (min)**

**B**


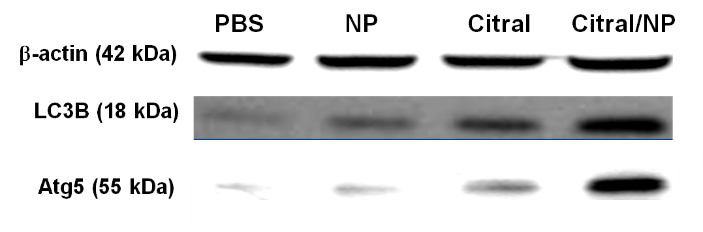


**D**

Treatment of SKOV-3 cells with citral increases acridine orange staining (A) and expression of LC3BII (B) suggesting occurrence of autophagy. Increase in ATG5 and LC3BII is also detected by western blotting (B).

Similar to ECC-1 and OVCAR-3, citral also induces an increase in intracellular oxygen radicals in SKOV-3 cells (C). Histograms shown in all figures are from flow cytometry experiments.

Expression of autophagy markers LC3B and Atg5 is also increased in 4T1 tumors excised from animals treated with vehicle control or with citral encapsulated PEG-PCL (NP) micelles (D).

**Supplementary File 4**

**p-eIF2α**

**eIF2α**

**30**

**60**

**120**

**0**


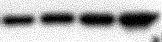

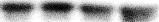


**ECC-1**

**Time (min)**


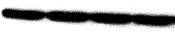


**Supplementary File 5**

Treatment of ECC-1 cells with citral (25 mM) results in an increase in phosphorylation of eIF2α, indicating occurrence of ER stress in these cells.

**β-Actin**


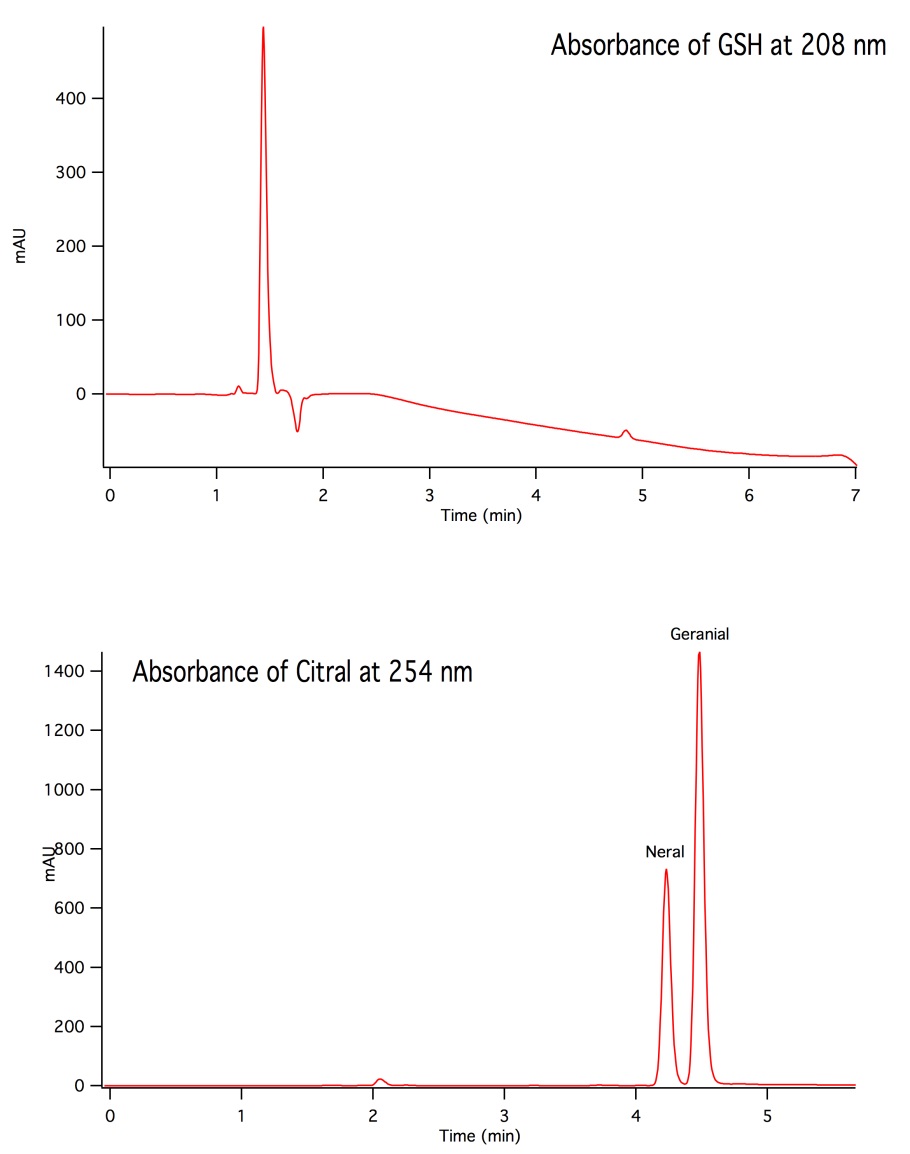

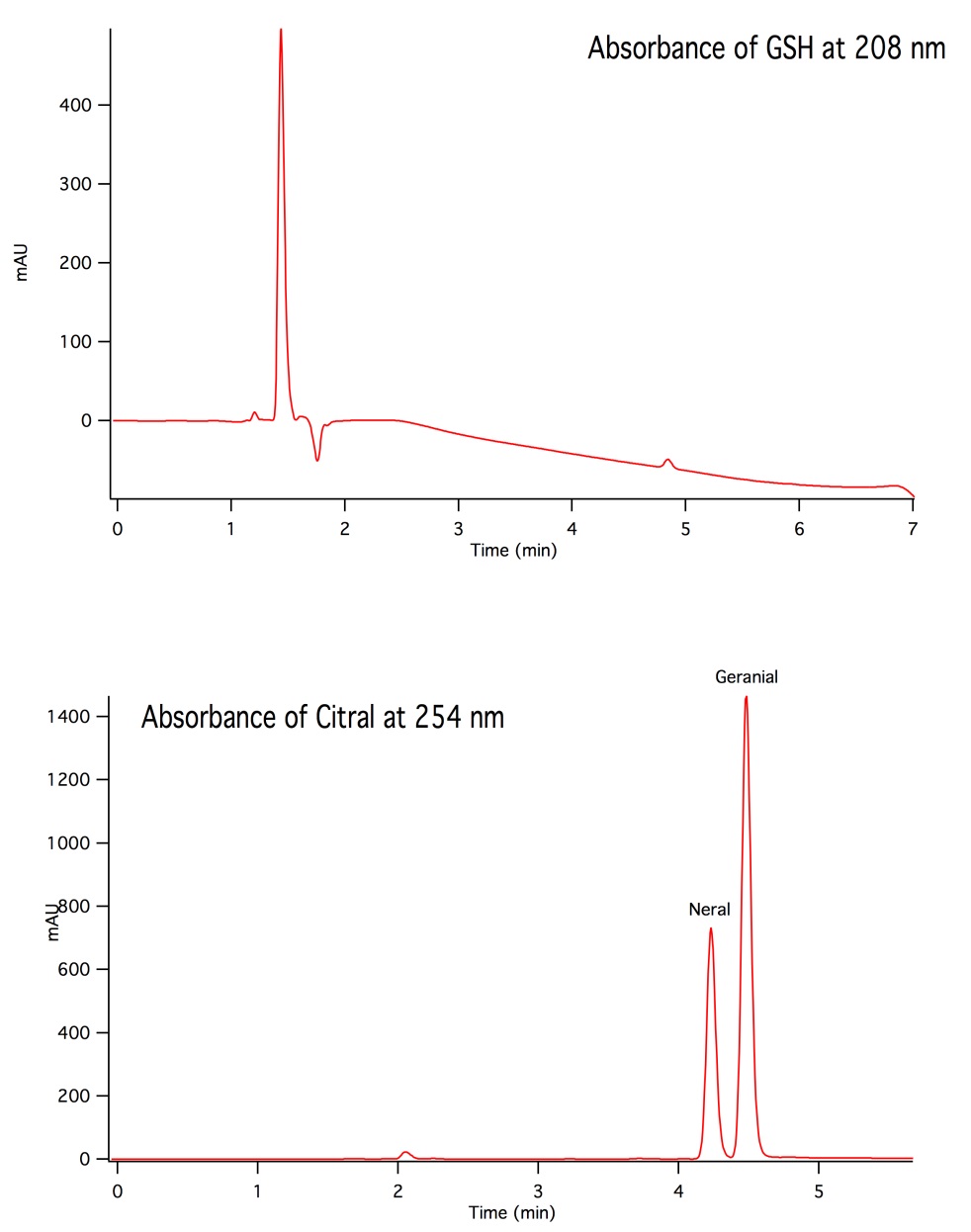

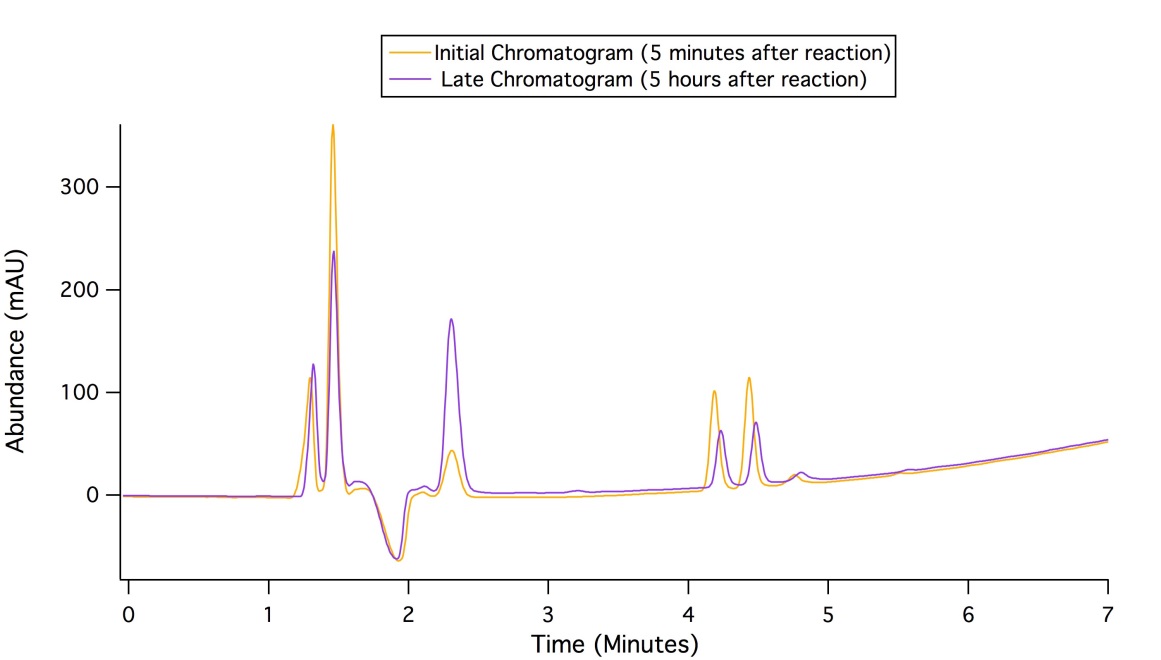


**Supplementary File 6**

GSH

Citral-GSH adduct

Neral

Geranial

Liquid chromatography of citral preparation used in the study

Liquid chromatography of glutathione

Liquid chromatography of reaction mixture after mixing citral with glutathione

**Supplementary File 6 (contd.)**

**Liquid chromatography with ultraviolet absorbance detection method for the Analysis of Citral, Glutathione and citral-glutathione adduct ’s Interaction with Glutathione**

The following method effectively and consistently separated GSH, neral, geranial, and the product into unique, identifiable peaks:

Stationary phase: Eclipse XDB C18 column

Column Dimensions: 4.6 X 150 mm

Column Particle Size: 5 μm

Column Temperature: 30**°** C

Flow Rate: 1 ml/min

Mobile phase:

‘A’ Solvent: Acidified water (0.10% acetic acid)

‘B’ Solvent: Methanol

Gradient:

T= 0 min 70% B Solvent 1 mL/min flow

T=5 min 90% B Solvent 1 mL/min flow

T=5.01 min 70% B Solvent 1 mL/min flow

Stoptime: 10 min

 Absorbance Detectors:

All (GSH, Citral, Product): 208 nm with 360, 50 reference

Citral isomers: 254 nm with 360, 50 reference

Injected Volume: 1 μL

 Samples tested with this method:

10 mM Citral in methanol

5.76 mM GSH in 4/7 methanol, 3/7 water solution

10 mM GSH in water

10 mM Citral solution + 10 mM GSH solution, mixed (pH ~ 3.5)

10 mM Citral solution + 10 mM GSH solution + 120 μL NaOH, mixed (pH ~10)
